# Understanding the Acid-Base Response to Respiratory Derangements: Finding, and Clinically Applying, the In Vivo Base Excess

**DOI:** 10.1097/CCE.0000000000001191

**Published:** 2024-12-16

**Authors:** Micah L. A. Heldeweg, Kenrick Berend, Patrick Schober, František Duška

**Affiliations:** 1 Department of Anesthesiology, Amsterdam University Medical Centers, Amsterdam, The Netherlands.; 2 Department of Anaesthesia and Intensive Care Medicine, The Third Faculty of Medicine, Charles University and FNKV University Hospital, Prague, Czech Republic.; 3 Department of Internal Medicine, Curaçao Medical Center, Willemstad, Curaçao.

**Keywords:** base deficit, buffer, carbon dioxide, metabolic, respiratory, resuscitation

## Abstract

**OBJECTIVES:**

To evaluate the base excess response during acute in vivo carbon dioxide changes.

**DESIGN:**

Secondary analysis of individual participant data from experimental studies.

**SETTING:**

Three experimental studies investigating the effect of acute in vivo respiratory derangements on acid-base variables.

**SUBJECTS:**

Eighty-nine (canine and human) carbon dioxide exposures.

**INTERVENTIONS:**

Arterial carbon dioxide titration through environmental chambers or mechanical ventilation.

**MEASUREMENTS AND MAIN RESULTS:**

For each subject, base excess was calculated using bicarbonate and pH using a fixed buffer power of 16.2. Analyses were performed using linear regression with arterial dioxide (predictor), base excess (outcome), and studies (interaction term). All studies show different baselines and slopes for base excess across carbon dioxide titrations methods. Individual subjects show substantial, and potentially clinically relevant, variations in base excess response across the hypercapnic range. Using a mathematical simulation of 10,000 buffer power coefficients we determined that a coefficient of 12.1 (95% CI, 9.1–15.1) instead of 16.2 facilitates a more conceptually appropriate in vivo base excess equation for general clinical application.

**CONCLUSIONS:**

In vivo changes in carbon dioxide leads to changes in base excess that may be clinically relevant for individual patients. A buffer power coefficient of 16.2 may not be appropriate in vivo and needs external validation in a range of clinical settings.

**KEY POINTS**

**Question:** Does in vivo carbon dioxide titration produce changes in (standard) base excess?

**Findings:** This secondary analysis of 89 individual exposures from three experimental studies on the acid-base response to acute respiratory derangements shows that base excess baseline and response is significantly different across studies using carbon dioxide titration. Using a fixed buffer power coefficient of 12.1 in the base excess equation improves its in vivo stability in healthy humans.

**Meaning:** Current base excess equations are not carbon dioxide invariant in vivo, but may be improved by adjusting the equation’s fixed buffer power coefficient.

Many acute care clinicians make clinical decisions based on acid-base indices such as base excess (BE) (1). Appropriate decision-making crucially depends on understanding parameters’ underlying mechanisms and limitations. BE was originally derived by laborious Pco_2_ tonometry and titration of the blood, but later replaced by the computationally simplified Van Slyke’s equation (2). In general, BE can be mathematically expressed as:


$$\begin{array}{l} BE=\left(H{\text{CO}}_{3}^{-}-24.8\right)+\left(pH-7.40\right)\cdot \beta \end{array}$$

β is the buffer power resisting pH change during Pco_2_ titration and depends on protein and hemoglobin levels. Earlier BE (blood) equations overestimated β by using in vitro measurements that did not accurately reflect the in vivo conditions. This concern was addressed by deriving a, theoretically, more stable standard BE (SBE) equation at a diluted and fixed extracellular hemoglobin concentration, yielding a β of 16.2 (2). In vitro stability of BE has since been confirmed by Morgan et al (3), but its in vivo stability remained a matter of debate and controversy (4). A previous meta-analysis of in vivo studies by Schlichtig et al (5) in *Critical Care Medicine* suggested that SBE is generally resilient to acute Pco_2_ changes. More recently, another review suggested that Pco_2_ variation does not cause clinically relevant in vivo SBE changes when β is set at 16.2 (6).

However, both reviews compiled data from in vivo human and canine experiments with divergent methodologies. The studies vary in subject species, timing, Pco_2_ titration method, infused IV drugs, and laboratory assays and results may therefore not be fully comparable. In this brief report, we reassess the literature to examine the stability of the in vivo BE across different studies and contribute original findings on the appropriate β for the in vivo BE equation.

## MATERIALS AND METHODS

This is a secondary analysis of in vivo experiments that evaluated the acid-base response across acute Pco_2_ variation. First, we evaluated the studies included in the previous meta-analysis and selected those that provided sufficient data to calculate the SBE. Then, we extracted all available individual participant data and compiled them in a single file with additional labels for studies and individual subject numbers. We calculated the SBE using the widely established SBE equation.


$$\begin{array}{l} SBE=\left(H{\text{CO}}_{3}^{-}-24.8\right)+\left(pH-7.40\right)\cdot 16.2 \end{array}$$

Then, we performed an analysis of covariance using SBE as the dependent variable, Pco_2_ as the independent variable, and tested the interaction between studies and Pco_2_.

Last, for the study in unanesthetized human volunteers, which was considered having the least confounders, we calculate the regression coefficient and extract individual changes in SBE across its Pco_2_ range. Conceptually, the relation between Pco_2_ and the true in vivo BE should have a regression coefficient of 0 (i.e., acute changes in Pco_2_ do not lead to changes in BE). Thus, using the Python computer programming language, we computed regression coefficients on the in vivo subject data provided in that study by simulating 10,000 different values for β, between 5 and 20. See **Supplemental Digital Content** for the full methods and Python script (http://links.lww.com/CCX/B443).

The same methodology was applied for the another SBE equation with an alternate fixed β coefficient, as previously published by Schlichtig et al (5):


$$\begin{array}{l} SBE=0.9287\cdot \left\{\left(H{\text{CO}}_{3}^{-}-24.8\right)+\left(pH-7.40\right)\cdot 14.83\right\} \end{array}$$

## RESULTS

Only three studies provided sufficient data to calculate SBE: one in unanesthetized human volunteers, one in mechanically ventilated and anesthetized patients undergoing elective surgery, and one in unanesthetized canines (5).

**Figure 1.**
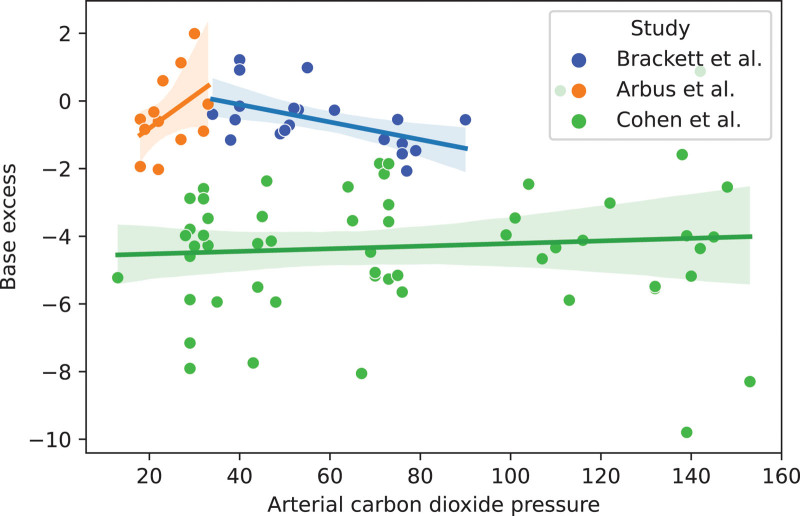
The changes in standard base excess during Pco_2_ titration across three studies. For full methodology and code, see Supplemental Digital Content (http://links.lww.com/CCX/B443).

**Figure 1** shows the SBE responses to Pco_2_ across 89 exposures in three study populations. We found significantly different intercepts (*p* < 0.001) and coefficients (*p* = 0.008) between the three studies (for full code and results, see Supplemental Digital Content, http://links.lww.com/CCX/B443).

The study by Brackett et al (7) introduces the least potential confounding by exclusively exposing unanesthetized volunteers to acutely ascending environmental levels of Pco_2_. In this study, the calculated SBE changes ranged from 0 to a clinically relevant –2.5 mmol/L, with linear regression coefficient of –0.025 mmol/L (95% CI, –0.05 to –0.0; *p* = 0.013) SBE per mm Hg of Pco_2_ (see Supplemental Digital Content for a figure containing the individual SBE responses to ascending Pco_2_ levels, http://links.lww.com/CCX/B443).

**Figure 2.**
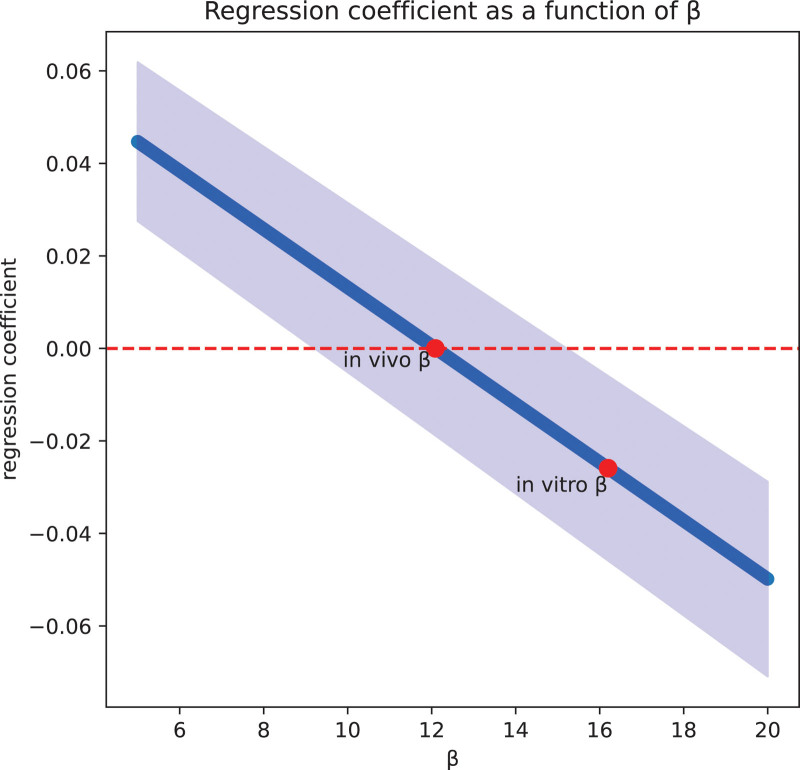
Linear regression coefficients as a function of 10,000 simulated β values between 5 and 20. Full methodology and code can be found in Supplemental Digital Content (http://links.lww.com/CCX/B443).

The value for β with the regression coefficient closest to zero was 12.1 (95% CI, 9.1–15.1) (**Fig. 2**; and for full code and results, see Supplemental Digital Content, http://links.lww.com/CCX/B443).

Using the SBE equation according to Schlichtig et al (5) did not produce different results (Supplemental Digital Content, http://links.lww.com/CCX/B443).

## DISCUSSION

The results of this study suggest that the baseline BE and response to acute in vivo Pco_2_ changes varies significantly between in vivo studies. Furthermore, in a selected study with least potential confounders, acute in vivo Pco_2_ variations over a hypercapnic range may produce clinically relevant changes in SBE when β is set at 16.2, but not when β is set at 12.1 (7). This is important because the in vivo acid-base response of the healthy human provides the most appropriate reference point to assess acid-base disorders. This is the first study that indicates, with existing data, that the currently widely used SBE equation and its (subjectively) fixed β are unstable in vivo even in healthy individuals.

Interestingly, the landmark study by Brackett et al (7) compared the in vivo results with in vitro experiments by titrating subjects’ pre-drawn arterial blood with varying concentrations of Pco_2_. The respective titration curves were found to be significantly different, reaffirming that the in vivo β is different from the in vitro β (7). Furthermore, considering the variability of SBE across individual subjects, it is likely that there is no single simplified beta-coefficient suitable for all individual patients. However, based on the limited data available, a coefficient of 12.1 appears to be better suited for general clinical application, with due regard for the wide 95% CI (9.1–15.1). Previous investigations have suggested alternative BE equations that are theoretically more stable across Pco_2_ variation, but these require more input variables and have not been validated in vivo (8).

Importantly, BE’s clinical application requires additional considerations. First, the degree of compensation, and thus baseline BE, crucially depends on the time course of the respiratory derangement (6). Second, changes in buffer equilibria due to disease states (e.g., abnormal proteins, hemoglobin, or renal failure) or as a result of therapy may directly alter β and lead to a deranged SBE (response) (8). A previous observational report found that acute (within 8–12 hr) Paco_2_ variation causes SBE changes in the opposite direction in critically ill patients (9). Furthermore, another study demonstrated how the induction of metabolic acidosis may directly alter β (10). Unfortunately, these factors and their respective impact, which should prompt more advanced acid-base investigations, are often unknown to clinicians at the point-of-care. Last, inconsistencies may also be introduced by site-specific reference ranges and technical analyzer limitations. However, those elements likely manifest as random noise, whereas the current β may produce a skewed signal in response to carbon dioxide.

The estimated β is 4.1 points lower than the currently employed β in SBE. In isolation, the clinical relevance of this difference may be minimal. However, SBE may deviate substantially in cases of large acute carbon dioxide changes, especially when compounded with individual variation in magnitude and other (abovementioned) confounders. In those cases, the clinician may erroneously suspect a superimposed metabolic acidosis, prompting unnecessary diagnostics or even preemptive therapy. Nonetheless, it should be emphasized that this is an exploratory study limited by precision (i.e., 95% CI) using existing data, and future prospective investigations are required to validate the appropriate β.

## CONCLUSIONS

BE is an elegant composite method to quantify metabolic acid-base derangements. This investigation demonstrates that in vivo changes in Pco_2_ leads to changes in SBE that may be clinically relevant for individual patients. Further validation is needed, but adjusting the β coefficient of SBE to 12.1 may improve its in vivo stability and its general clinical applicability. Furthermore, BE may carry inaccuracies of different magnitudes depending stage of compensation and state of buffer equilibria, which are frequently unknown at the point-of-care. Although single imprecisions may not produce clinically relevant issues, multiple coexisting and compounding uncertainties, especially in critically ill patients, should prompt clinicians to exercise caution. Robust experimental data is needed to find a universally, and conceptually, accurate BE and gain deeper understanding of its limitations.

## Supplementary Material


