# Supplementary material for: Understanding the Acid-Base Response to Respiratory Derangements: Finding, and Clinically Applying, the In Vivo Base Excess
Source: Crit Care Explor. 2024 Dec 16;6(12):e1191. doi: 10.1097/CCE.0000000000001191 (PMC11651497; doi:10.1097/CCE.0000000000001191)
Supplement: Supplementary file 1 [file cc9-6-e1191-s001.pdf]

**Supplemental Digital Content**

All data was extracted from studies included in the meta-analyses by Zadek et al. and Schlichtig et al. that concern acute respiratory derangements and had sufficient data available to calculate the base excess.[1,2] The following data was extracted from each:

1. Brackett and colleagues: Table 1. Acid-Base Parameters of Plasma during Acute Hypercapnia. Data from all subjects at control, 7 percent, and 10 percent were extracted[3]
2. Arbus and colleagues: Table 2. Plasma Acid-Base Changes at 10 Minutes of hyperventilation. Data from all subjects at control and hyperventilation were extracted[4]
3. Cohen and colleagues: Table 2. Acid-base parameters of plasma during titration of the dog with carbon dioxide. Only used data from experiment A and B, ascending carbon dioxide titration[5]
4. Jarahevi and colleagues: insufficient data could be extracted to calculated base excess[6]

All extracted data was compiled in a single file with labels for different studies, hypocapnia or hypercapnia experiments, species, and sequential subject number. The analysis was performed in Jupyter Notebook using the Python programming language (version 3.8).[7] All code scripts and results are below using single-line comments (green, indicated by '#') to describe the method used in plain language.

The calculation of standard base excess was performed using the widely established standard base excess equation (as also utilized by Zadek and colleagues):

$$\boldsymbol{Standard\ base\ excess} = (\boldsymbol{HCO_3^-} - \boldsymbol{24.8}) + (\boldsymbol{pH} - \boldsymbol{7.40}) \cdot \boldsymbol{16.2}$$

All linear regression analyses were performed using the ordinary least squares method. We compared the intercepts and regression coefficients between study types by adding an interaction term (pco2:study) to the model.

We separately investigated the individual cases by Brackett and colleagues because there was no additional use of anaesthetics, intravenous fluids, different species, mechanical ventilation, which can all be considered potential confounding factors. For the individual cases from the study by Brackett and colleagues we performed the same analyses. Figure S1 shows the individual changes in base excess during carbon dioxide titration in the study by Brackett and colleagues.

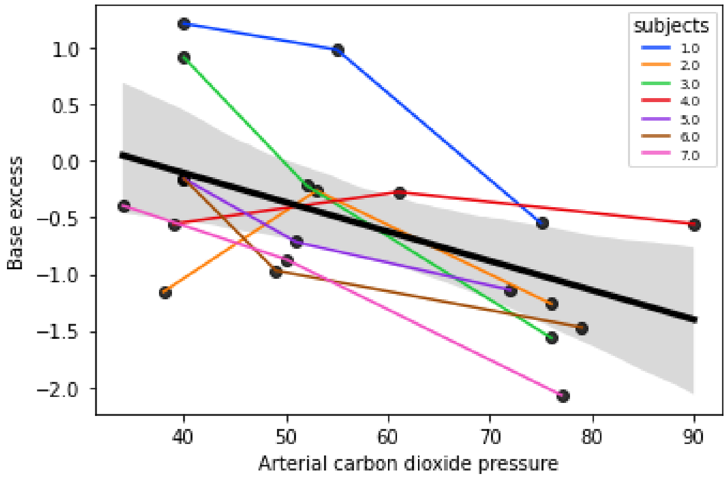


**Figure S1.** the individual changes in base excess during carbon dioxide titration in the study by Brackett and colleagues

Additionally, we calculated the difference in standard base excess between the normocapnic and 10% carbon dioxide hypercapnic atmospheres.

Then, we created a list with 10,000 (uniformly spaced) values of β between 5 and 20. For each value of β in the base excess equation we calculated a new regression coefficient for carbon dioxide (predictor) and base excess (outcome) on the dataset by Brackett and colleagues. We performed this using the iterative loop functionality in Python.

$$\boldsymbol{base\ excess} = (\boldsymbol{HCO_3^-} - \mathbf{24.8}) + (\boldsymbol{pH} - \mathbf{7.40}) \cdot \boldsymbol{\beta}$$

Afterwards, we located the coefficient and confidence interval closest to zero. We obtained the β associated with these values to represent the *in vivo* buffer power. This value was, as is intrinsic to the statistical method, the same as the value of β associated with the p-value closest to one. Figure S2 shows the p-values associated with each value of β.

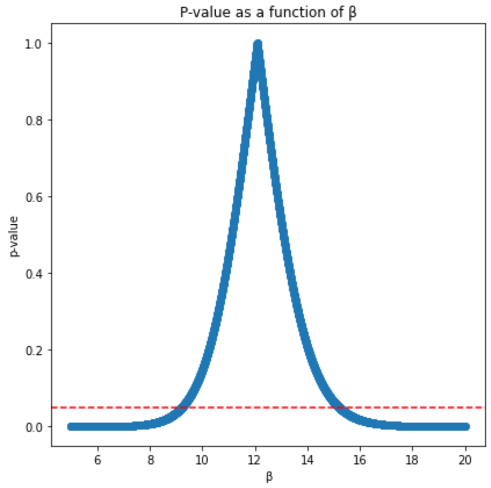


**Figure S2.** P-values associated with the linear regression analysis using different values for β

The confidence interval of β was 9.1 to 15.1. Its limited precision is caused by the low sample size of the original investigation. Moreover, the carbon dioxide range can generally be considered hypercapnic. Last, the original investigation by Brackett and colleagues shows that there may be a non-linear relationship between carbon dioxide and ex vivo buffer capacity. Both are limitations to this investigation and illustrate the need for further research and clinical caution.

The alternative calculation of base excess, according to Schlichtig et al., was also performed according to the following equation[2]:

$$\boldsymbol{Standard\ base\ excess} = \mathbf{0.9287} \cdot \{(\boldsymbol{HCO_3^-} - \mathbf{24.8}) + (\boldsymbol{pH} - \mathbf{7.40}) \cdot \mathbf{14.83}\}$$

The results for the latter can be found at the last segment of the Python script below. The results were not different from the calculations made with the more widely used standard base excess as used by Zadek et al.

**References**


1. Zadek F, Danieli A, Brusatori S, Giosa L, Krbec M, Antolini L, Fumagalli R, Langer T. Combining the Physical-Chemical Approach with Standard Base Excess to Understand the Compensation of Respiratory Acid-Base Derangements: An Individual Participant Meta-analysis Approach to Data from Multiple Canine and Human Experiments. Anesthesiology. 2024 Jan 1;140(1):116-125. doi: 10.1097/ALN.0000000000004751. PMID: 37616330.
2. Schlichtig R, Grogono AW, Severinghaus JW. Human PaCO2 and standard base excess compensation for acid-base imbalance. Crit Care Med. 1998 Jul;26(7):1173-9. doi: 10.1097/00003246-199807000-00015. PMID: 9671365.
3. Brackett NC Jr, Cohen JJ, Schwartz WB. Carbon dioxide titration curve of normal man. Effect of increasing degrees of acute hypercapnia on acid-base equilibrium N Engl J Med. 1965 Jan 7;272:6-12. doi: 10.1056/NEJM196501072720102. PMID: 14219220.
4. Javaheri S, Weyne J, Demeester G, Leusen I. Effects of acetazolamide on ionic composition of cisternal fluid during acute respiratory acidosis. J Appl Physiol Respir Environ Exerc Physiol. 1984 Jul;57(1):85-91. doi: 10.1152/jappl.1984.57.1.85. PMID: 6469795.
5. Cohen JJ, Brackett NC Jr, Schwartz WB. The nature of the carbon dioxide titration curve in the normal Dog. J Clin Invest. 1964 May;43(5):777-86. doi: 10.1172/JCI104964. PMID: 14169507; PMCID: PMC289557.
6. Arbus GS, Herbert LA, Levesque PR, Etsten BE, Schwartz WB. Characterization and clinical application of the "significance band" for acute respiratory alkalosis. N Engl J Med. 1969 Jan 16;280(3):117-23. doi: 10.1056/NEJM196901162800301. PMID: 5782512.
7. Van Rossum G, Drake Jr FL. Python reference manual. Centrum voor Wiskunde en Informatica Amsterdam; 1995

In [1]:

```python
# Importing all libraries
import numpy as np
import pandas as pd
import seaborn as sns
import matplotlib.pyplot as plt
import statsmodels.api as sm
import statsmodels.formula.api as smf
from sklearn.linear_model import LinearRegression
from sklearn.metrics import log_loss
import math
# Importing an Excel file with all the data from acute experiments.
df = pd.read_excel('acute ascending experiments.xlsx')
# The excel files contains data from three papers:
# Brackett and colleagues: Table 1. Acid-Base Paramaters of Plasma during Acute Hypercapnia
# Data from all subjects at control, 7 percent, and 10 percent were extracted
# Arbus and colleagues: Table 2. Plasma Acid-Base Changes at 10 Minutes of hyperventilation
# Data from all subjects at control and hyperventilation were extracted
# Cohen and colleagues: Table 2. Acid-base parameters of plasma during titration of the dog with carbon dioxide
# Only used data from experiment A and B, ascending carbon dioxide titration
# We were unable to extract sufficient information from the article by Jahaveri and colleagues to calculate SBE
```

In [2]:

```python
#Converting all the data to usable formats
# Convert 'hydrogen' column to numeric
df['hydrogen_num'] = pd.to_numeric(df['hydrogen'], errors='coerce')
# Convert nanomoles per liter to moles per liter
df['h+'] = df['hydrogen_num'] / 1e9
# Calculate pH using the negative logarithm
df['pH'] = -1 * (df['h+'].apply(math.log10))
# calculate SBE
df["SBE"] = (df['bicarbonate'] - 24.8) + (16.2*(df['pH'] - 7.40))
df["SBE_schlichtig"] = 0.9287 * (df['bicarbonate'] - 24.4) + (14.83*(df['pH'] - 7.40))
# Calculate fraction of inspired CO2
df['atmospheric CO2'] = (df['atmosphere']/100) * 101.33
# create a seperate dataframe for the brackett study
df_brackett = df[df['study'] == 0]
# Show an example of the dataframe
df
```

Out[2]:

| | humans | study | acidosis | subject | atmosphere | pco2 | hydrogen | bicarbonate | hydrogen_num | h+ | pH | SBE | SBE_schlichtig |
|---|---|---|---|---|---|---|---|---|---|---|---|---|---|
| **0** | 0.0 | 0 | 0.0 | 1.0 | 1.0 | 40.0 | 37.0 | 25.5 | 37.0 | 3.700000e-08 | 7.431798 | 1.215132 | 1.493138 |
| **1** | 0.0 | 0 | 0.0 | 2.0 | 1.0 | 38.0 | 39.0 | 23.5 | 39.0 | 3.900000e-08 | 7.408935 | -1.155247 | -0.703318 |
| **2** | 0.0 | 0 | 0.0 | 3.0 | 1.0 | 40.0 | 37.0 | 25.2 | 37.0 | 3.700000e-08 | 7.431798 | 0.915132 | 1.214528 |
| **3** | 0.0 | 0 | 0.0 | 4.0 | 1.0 | 39.0 | 39.0 | 24.1 | 39.0 | 3.900000e-08 | 7.408935 | -0.555247 | -0.146098 |
| **4** | 0.0 | 0 | 0.0 | 5.0 | 1.0 | 40.0 | 39.0 | 24.5 | 39.0 | 3.900000e-08 | 7.408935 | -0.155247 | 0.225382 |
| **...** | ... | ... | ... | ... | ... | ... | ... | ... | ... | ... | ... | ... | ... |
| **84** | NaN | 2 | NaN | NaN | 18.0 | 139.0 | 117.0 | 28.4 | 117.0 | 1.170000e-07 | 6.931814 | -3.984611 | -3.228396 |
| **85** | NaN | 2 | NaN | NaN | 18.0 | 145.0 | 121.0 | 28.6 | 121.0 | 1.210000e-07 | 6.917215 | -4.021123 | -3.259167 |
| **86** | NaN | 2 | NaN | NaN | 18.0 | 153.0 | 143.0 | 25.5 | 143.0 | 1.430000e-07 | 6.844664 | -8.296444 | -7.214063 |
| **87** | NaN | 2 | NaN | NaN | 18.0 | 142.0 | 120.0 | 28.2 | 120.0 | 1.200000e-07 | 6.920819 | -4.362736 | -3.577198 |
| **88** | NaN | 2 | NaN | NaN | 18.0 | 148.0 | 118.0 | 29.9 | 118.0 | 1.180000e-07 | 6.928118 | -2.544489 | -1.890160 |

89 rows × 14 columns

In [3]:

```python
# Create a figure containing all different subtypes using sns.regplot
g = sns.scatterplot(data=df, x='pco2', y='SBE', hue='study', palette='bright')
sns.regplot(x='pco2', y='SBE', data=df[df['study'] == 0],scatter=False)
sns.regplot(x='pco2', y='SBE', data=df[df['study'] == 1],scatter=False)
sns.regplot(x='pco2', y='SBE', data=df[df['study'] == 2],scatter=False)
# Create figure labeling
study_labels = {0: 'Brackett et al.', 1: 'Arbus et al.', 2: 'Cohen et al.'}
plt.xlabel('Arterial carbon dioxide pressure')
plt.ylabel('Base excess')
handles, labels = g.get_legend_handles_labels()
plt.legend(handles, [f"{study_labels[int(label)]}" for label in labels], title='Study', loc='upper right')
```

Out[3]:

```
<matplotlib.legend.Legend at 0x24acbdf5c40>
```

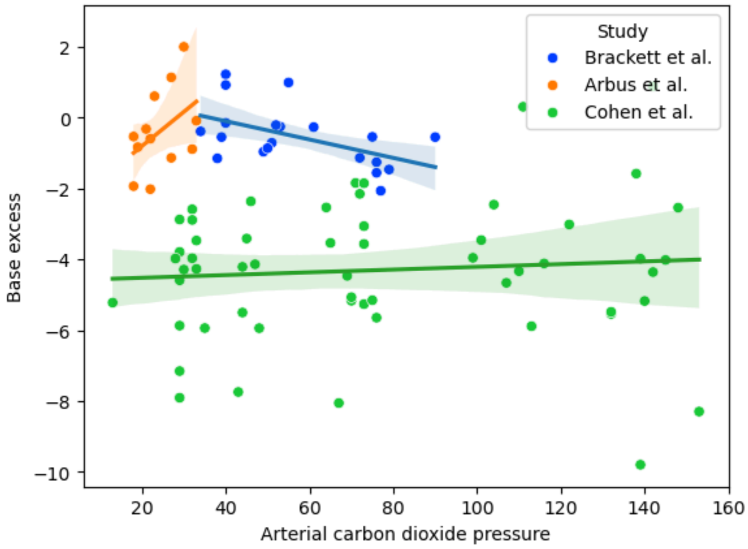


In [4]:
```
# analysis of covariance investigating the relationship between pco2 and SBE using study as a covariating factor
# using an ordinary least squares method to fit the model
model = smf.ols('SBE ~ pco2 * study', data=df).fit()
print(model.summary())
### interpretation of results
# intercept: baseline value of 'SBE' when 'pco2' is zero and the study group is the first reference group (brackett)
# pco2: slope of the regression line for 'pco2' and 'SBE' when 'study' is at its first reference level (brakett)
# study: differences in intercepts between each study group and the reference group (brackett)
# pco2:study interaction term: differences in regression coefficients between different levels of the 'study' variable
```

```
                            OLS Regression Results
==============================================================================
Dep. Variable:                    SBE   R-squared:                       0.526
Model:                            OLS   Adj. R-squared:                  0.509
Method:                 Least Squares   F-statistic:                     31.45
Date:                Sat, 10 Aug 2024   Prob (F-statistic):           8.95e-14
Time:                        19:25:06   Log-Likelihood:                -173.87
No. Observations:                  89   AIC:                             355.7
Df Residuals:                      85   BIC:                             365.7
Df Model:                           3
Covariance Type:            nonrobust
==============================================================================
                 coef    std err          t      P>|t|      [0.025      0.975]
------------------------------------------------------------------------------
Intercept      2.8539      1.075      2.654      0.010       0.716       4.992
pco2          -0.0550      0.019     -2.872      0.005      -0.093      -0.017
study         -3.5623      0.631     -5.646      0.000      -4.817      -2.308
pco2:study     0.0280      0.010      2.706      0.008       0.007       0.048
==============================================================================
Omnibus:                        6.645   Durbin-Watson:                   1.439
Prob(Omnibus):                  0.036   Jarque-Bera (JB):                9.901
Skew:                          -0.223   Prob(JB):                      0.00708
Kurtosis:                       4.572   Cond. No.                     1.03e+03
==============================================================================

Notes:
[1] Standard Errors assume that the covariance matrix of the errors is correctly specified.
[2] The condition number is large, 1.03e+03. This might indicate that there are
strong multicollinearity or other numerical problems.
```

In [5]:
```
# Plot a figure for the study by Brackett and colleagues
sns.lineplot(data=df_brackett, x='pco2', y='SBE', hue='subject', palette='bright')
sns.regplot(x='pco2', y='SBE', data=df_brackett, scatter=True, color='black', line_kws={'linewidth':3.5})
# Create labelling for the figure
plt.legend(title='subjects', fontsize='x-small')
plt.xlabel('Arterial carbon dioxide pressure')
plt.ylabel('Base excess')
# Calculate the delta SBE between first experiment (atmosphere 1) and the last experiment (atmosphere 10)
df_pivot = df_brackett.pivot(index='subject', columns='atmosphere', values='SBE')
df_pivot['delta_SBE'] = df_pivot[10] - df_pivot[1]
df_delta = df_pivot.reset_index()
print(df_delta['delta_SBE'].describe())
```

```
count    7.000000
mean    -1.189173
std      0.900705
min     -2.477544
25%     -1.719891
50%     -1.314109
75%     -0.546017
max     -0.000746
Name: delta_SBE, dtype: float64
```

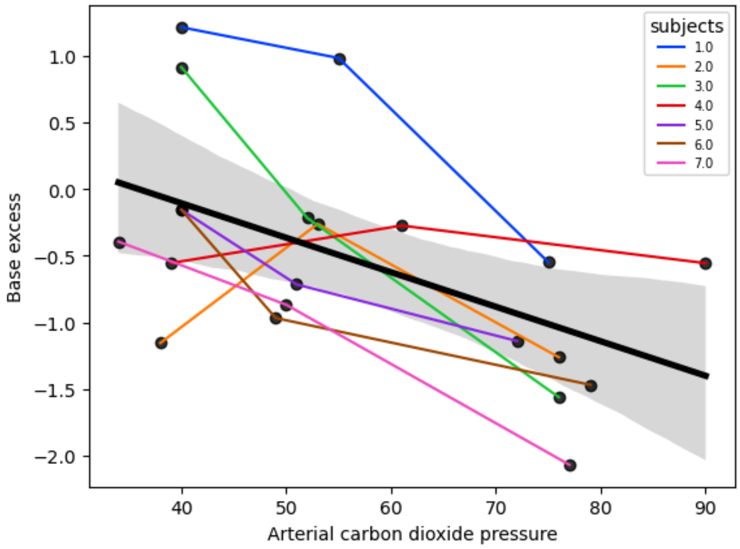


In [6]:
```
# Calculate the linear regression coefficient for the relation between pCO2 (independent) and SBE (dependent)
# Using a OLS linear regression model
predictor = sm.add_constant(df_brackett[['pco2']])
outcome = df_brackett['SBE']
model = sm.OLS(outcome, predictor).fit()
print(model.summary())
```

```
                            OLS Regression Results
==============================================================================
Dep. Variable:                    SBE   R-squared:                       0.281
Model:                            OLS   Adj. R-squared:                  0.243
Method:                 Least Squares   F-statistic:                     7.417
Date:                Sat, 10 Aug 2024   Prob (F-statistic):             0.0135
Time:                        19:25:14   Log-Likelihood:                -22.021
No. Observations:                  21   AIC:                             48.04
Df Residuals:                      19   BIC:                             50.13
Df Model:                           1
Covariance Type:            nonrobust
==============================================================================
                 coef    std err          t      P>|t|      [0.025      0.975]
------------------------------------------------------------------------------
const          0.9309      0.561      1.660      0.113      -0.243       2.105
pco2          -0.0259      0.010     -2.723      0.013      -0.046      -0.006
==============================================================================
Omnibus:                        1.806   Durbin-Watson:                   2.385
Prob(Omnibus):                  0.405   Jarque-Bera (JB):                1.439
Skew:                           0.609   Prob(JB):                        0.487
Kurtosis:                       2.596   Cond. No.                         209.
==============================================================================

Notes:
[1] Standard Errors assume that the covariance matrix of the errors is correctly specified.
```

In [ ]:
```
# Calculating regression coefficients and p-values for different β values in the SBE equation
# Constants to be selected (between 5 and 20, with 10,000 repetitions)
constants = np.linspace(5, 20, 10000)
# Placeholders
regression_coefficients = []
p_values = []
conf_intervals_lower = []
conf_intervals_upper = []
# loop the calculation of regression coefficients across all values of β in the constants
for constant in constants:
    # calculated base excess
    df_brackett['cBE'] = (df_brackett['bicarbonate'] - 24.8) + (constant*(df_brackett['pH'] - 7.40))
    # Add a constant to the predictor for the intercept
    predictor = sm.add_constant(df_brackett[['pco2']])
    outcome = df_brackett['cBE']
    # Create a model
    model = sm.OLS(outcome, predictor)
    results = model.fit()
    # get the regression coefficient, 95% CI, p-values
    regression_coefficient = results.params['pco2']
    p_value = results.pvalues['pco2']
    conf_interval = results.conf_int().loc['pco2']
    # append the values
    regression_coefficients.append(regression_coefficient)
    p_values.append(p_value)
    conf_intervals_lower.append(conf_interval[0])
    conf_intervals_upper.append(conf_interval[1])
# Make a new plottable dataframe to examine
results = pd.DataFrame({
    'buffer_power': constants,
    'regression_coefficient': regression_coefficients,
    'p_value': p_values,
    'conf_interval_lower': conf_intervals_lower,
```

```
    'conf_interval_upper': conf_intervals_upper
})
```

In [8]:
```
# Find the buffer power that gives the regression coefficient closes to 0 and p-value closest to 1
p_value_closest_to_1 = results.iloc[(results['p_value'] - 1).abs().argmin()]['buffer_power']
coefficient_closest_to_0 = results.iloc[(results['regression_coefficient']).abs().argmin()]['buffer_power']
# Find the buffer power for the confidence interval around the coefficient
target_neg = -0.018796
target_pos = 0.018818
closest_to_neg = results.iloc[(results['regression_coefficient'] - target_neg).abs().idxmin()]['buffer_power']
closest_to_pos = results.iloc[(results['regression_coefficient'] - target_pos).abs().idxmin()]['buffer_power']
print(f'buffer power at a p-value of 1: {p_value_closest_to_1}')
print(f'buffer power a regression coefficient of 0: {coefficient_closest_to_0}')
print(f'confidence interval of the buffer power: {closest_to_pos} to {closest_to_neg}')
```

```
buffer power at a p-value of 1: 12.088208820882087
buffer power a regression coefficient of 0: 12.088208820882087
confidence interval of the buffer power: 9.104410441044104 to 15.07050705070507
```

In [9]:
```
plt.figure(figsize=(6, 6))
# Plot results dataframe with values for the regression coefficient
sns.scatterplot(data=results, x='buffer_power', y= 'regression_coefficient', edgecolor=None)
plt.fill_between(results['buffer_power'], results['conf_interval_lower'], results['conf_interval_upper'], color='b', alpha=0.2, label
plt.axhline(y=0, color='r', linestyle='--', label='No relationship (Coefficient = 0)')
plt.xlabel('β')
plt.ylabel('regression coefficient')
plt.title('Regression coefficient as a function of β')
# plot markers for the appropriate in vivo and ex vivo β
plt.plot(16.2, -0.0259, 'ro', markersize=8)
plt.annotate(f'in vitro β', xy=(16.2, -0.028), xytext=(16.2 - 2.5, -0.028 - 0.0035))
plt.plot(12.087, 0.0, 'ro', markersize=8)
plt.annotate(f'in vivo β', xy=(12.087, 0.0), xytext=(12.087 - 2.5, -0.000 - 0.006))
plt.savefig("Fig1.pdf", dpi=300, bbox_inches='tight')
```

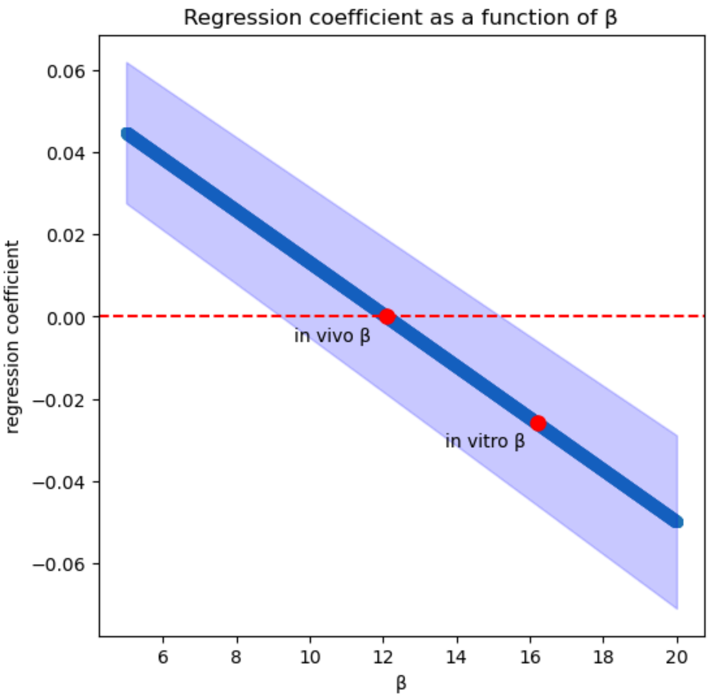


In [10]:
```
plt.figure(figsize=(6, 6))
# Plot p-values
sns.scatterplot(data=results, x='buffer_power', y= 'p_value', edgecolor=None)
# Plot a line at the 'significant' p-value of β
plt.axhline(y=0.05, color='r', linestyle='--', label='No relationship (Coefficient = 0)')
plt.xlabel('β')
plt.ylabel('p-value')
plt.title('P-value as a function of β')
plt.tight_layout()
```

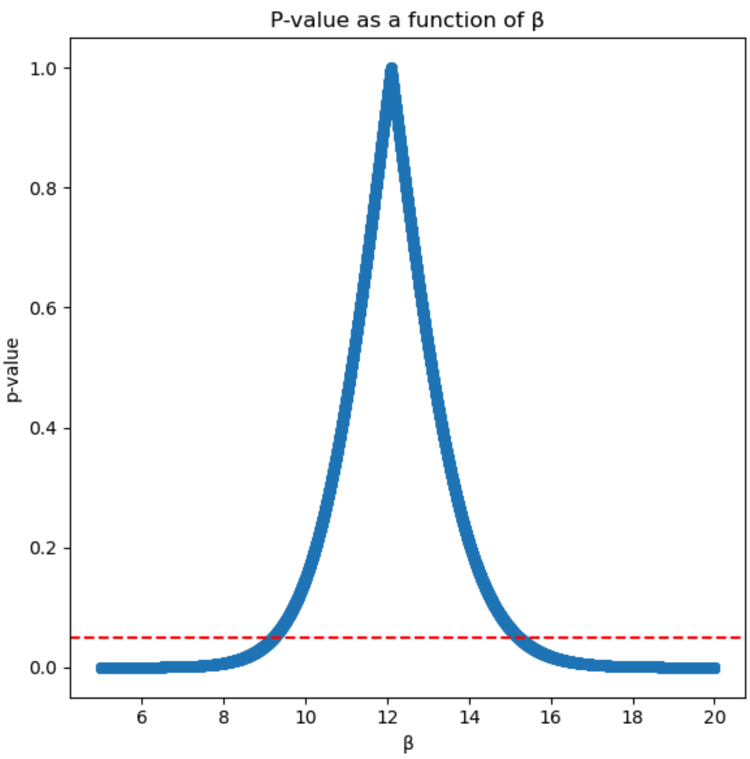


In [11]:
```python
# All variations tested with the simplified SBE equation as used by Schlichtig et al.

# Create a figure containing all different subtypes using sns.regplot
g = sns.scatterplot(data=df, x='pco2', y='SBE_schlichtig', hue='study', palette='bright')
sns.regplot(x='pco2', y='SBE_schlichtig', data=df[df['study'] == 0],scatter=False)
sns.regplot(x='pco2', y='SBE_schlichtig', data=df[df['study'] == 1],scatter=False)
sns.regplot(x='pco2', y='SBE_schlichtig', data=df[df['study'] == 2],scatter=False)
# Create figure labeling
study_labels = {0: 'Brackett et al.', 1: 'Arbus et al.', 2: 'Cohen et al.'}
plt.xlabel('Arterial carbon dioxide pressure')
plt.ylabel('Base excess according to Schlichtig')
handles, labels = g.get_legend_handles_labels()
plt.legend(handles, [f"{study_labels[int(label)]}" for label in labels], title='Study', loc='upper right')
```

Out[11]: <matplotlib.legend.Legend at 0x24accf17160>

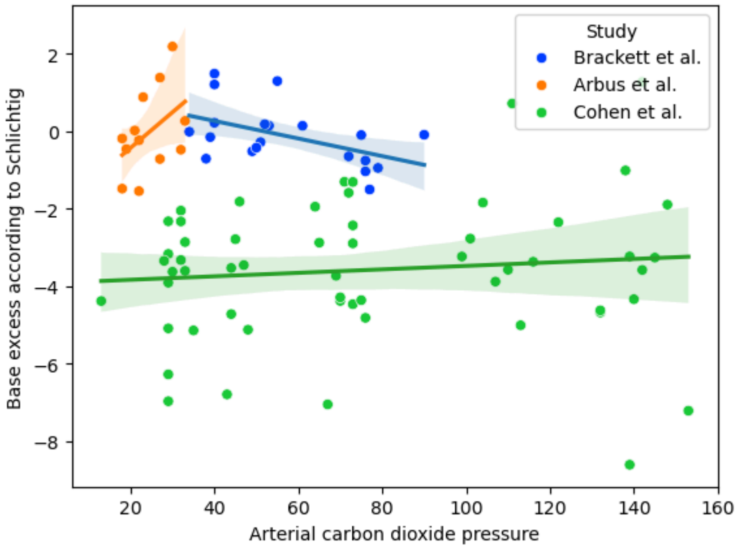


In [12]:
```python
# The prediction model with interaction term for SBE calculated according to Schlichtig et al.
model = smf.ols('SBE_schlichtig ~ pco2 * study', data=df).fit()
print(model.summary())
```

```
                            OLS Regression Results
==============================================================================
Dep. Variable:          SBE_schlichtig   R-squared:                       0.522
Model:                             OLS   Adj. R-squared:                  0.505
Method:                  Least Squares   F-statistic:                     30.89
Date:                 Sat, 10 Aug 2024   Prob (F-statistic):           1.34e-13
Time:                         19:32:40   Log-Likelihood:                -166.97
No. Observations:                   89   AIC:                             341.9
Df Residuals:                       85   BIC:                             351.9
Df Model:                            3
Covariance Type:             nonrobust
==============================================================================
                 coef    std err          t      P>|t|      [0.025      0.975]
------------------------------------------------------------------------------
Intercept      2.9374      0.995      2.951      0.004       0.959       4.916
pco2          -0.0493      0.018     -2.780      0.007      -0.085      -0.014
study         -3.2782      0.584     -5.615      0.000      -4.439      -2.117
pco2:study     0.0255      0.010      2.670      0.009       0.007       0.045
==============================================================================
Omnibus:                        6.745   Durbin-Watson:                   1.444
Prob(Omnibus):                  0.034   Jarque-Bera (JB):               10.141
Skew:                          -0.226   Prob(JB):                      0.00628
Kurtosis:                       4.591   Cond. No.                     1.03e+03
==============================================================================

Notes:
[1] Standard Errors assume that the covariance matrix of the errors is correctly specified.
[2] The condition number is large, 1.03e+03. This might indicate that there are
strong multicollinearity or other numerical problems.
```

In [13]:

```
# Plot a figure for the study by Brackett and colleagues for SBE calculated according to Schlichtig et al.
sns.lineplot(data=df_brackett, x='pco2', y='SBE_schlichtig', hue='subject', palette='bright')
sns.regplot(x='pco2', y='SBE_schlichtig', data=df_brackett, scatter=True, color='black', line_kws={'linewidth':3.5})
# Create labelling for the figure
plt.legend(title='subjects', fontsize='x-small')
plt.xlabel('Arterial carbon dioxide pressure')
plt.ylabel('Base excess')
# Calculate the delta SBE_schlichtig between first experiment (atmosphere 1) and the last experiment (atmosphere 10)
df_pivot = df_brackett.pivot(index='subject', columns='atmosphere', values='SBE_schlichtig')
df_pivot['delta_SBE_schlichtig'] = df_pivot[10] - df_pivot[1]
df_delta = df_pivot.reset_index()
print(df_delta['delta_SBE_schlichtig'].describe())
```

```
count    7.000000
mean    -1.049752
std      0.836047
min     -2.245468
25%     -1.539283
50%     -1.167154
75%     -0.458711
max      0.060350
Name: delta_SBE_schlichtig, dtype: float64
```

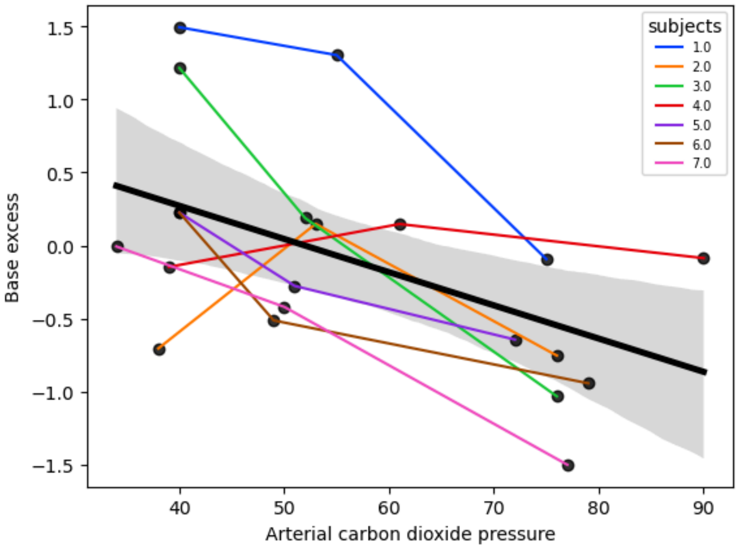


In [14]:

```
# Calculate the linear regression coefficient for the relation between pCO2 (independent) and SBE (dependent)
# SBE calculated according to Schlichtig et al.
# Using a OLS linear regression model
predictor = sm.add_constant(df_brackett[['pco2']])
outcome = df_brackett['SBE_schlichtig']
model = sm.OLS(outcome, predictor).fit()
print(model.summary())
```

```
                            OLS Regression Results
==============================================================================
Dep. Variable:         SBE_schlichtig   R-squared:                       0.259
Model:                            OLS   Adj. R-squared:                  0.220
Method:                 Least Squares   F-statistic:                     6.649
Date:                Sat, 10 Aug 2024   Prob (F-statistic):             0.0184
Time:                        19:33:49   Log-Likelihood:                -20.399
No. Observations:                  21   AIC:                             44.80
Df Residuals:                      19   BIC:                             46.89
Df Model:                           1
Covariance Type:            nonrobust
==============================================================================
                 coef    std err          t      P>|t|      [0.025      0.975]
------------------------------------------------------------------------------
const          1.1808      0.519      2.275      0.035       0.094       2.267
pco2          -0.0227      0.009     -2.578      0.018      -0.041      -0.004
==============================================================================
Omnibus:                        1.792   Durbin-Watson:                   2.384
Prob(Omnibus):                  0.408   Jarque-Bera (JB):                1.427
Skew:                           0.606   Prob(JB):                        0.490
Kurtosis:                       2.597   Cond. No.                         209.
==============================================================================

Notes:
[1] Standard Errors assume that the covariance matrix of the errors is correctly specified.
```
